# Cell-mediated targeting drugs delivery systems

**DOI:** 10.1080/10717544.2020.1831103

**Published:** 2020-10-23

**Authors:** Hongli Yu, Zhihong Yang, Fei Li, Lisa Xu, Yong Sun

**Affiliations:** a Department of Pharmacology, School of Pharmacy, Qingdao University, Qingdao, China; b Department of Pharmacy, Affiliated Hospital of Qingdao University, Qingdao, China; c School of Public Health, Qingdao University, Qingdao, China

**Keywords:** Cell-carriers, targeted drug delivery, immune cells, circulating cells, stem cells, targeting

## Abstract

Drug delivery systems have shown tremendous promise to improve the diagnostic and therapeutic effects of drugs due to their special property. Targeting tissue damage, tumors, or drugs with limited toxicity at the site of infection is the goal of successful pharmaceuticals. Targeted drug delivery has become significantly important in enhancing the pharmaceutical effects of drugs and reducing their side effects of therapeutics in the treatment of various disease conditions. Unfortunately, clinical translation of these targeted drug delivery system mechanisms faces many challenges. At present, only a few targeted drug delivery systems can achieve high targeting efficiency after intravenous injection, even though numerous surface markers and targeting approaches have been developed. Thus, cell-mediated drug-delivery targeting systems have received considerable attention for their enhanced therapeutic specificity and efficacy in the treatment of the disease. This review highlights the recent advances in the design of the different types of cells that have been explored for cell-mediated drug delivery and targeting mechanisms. A better understanding of cell biology orientation and a new generation of delivery strategies that utilize these endogenous approaches are expected to provide better solutions for specific site delivery and further facilitate clinical translation.

## 1. Introduction

Small molecule drugs lack tissue and organ specificity, suffer from rapid *in vivo* body clearance, and are often accompanied by many side effects, especially chemotherapeutic agents, which are usually highly toxic (Ayer & Klok, 2017). In the past decades, drug delivery systems (DDS) have been used as one of the most promising strategies to address this issue. The use of a carrier system ensures can help enhance the specificity and safety of the therapeutic, diagnostic, or prophylactic agents and to further improve its efficacy (Ma, Gao, et al., 2020). The key functions of these carriers include prolonging the half-life of drugs, effectively targeting the target sites of therapeutic drugs, thus minimizing the impact on non-target tissues (Chi et al., 2020). However, because traditional DDS can not achieve real targeted therapies and personalized medical treatment and cannot meet the growing needs of modern medicine (Su et al., 2015). Thus, develop a new type of DDS with truly specific targeting is a daunting challenge for modern medicine (Ma, Cao, et al., 2020).

In recent years, cell-mediated DDS has become a promising strategy to address the above challenges (Ma, Song, et al., 2020; Shen et al., 2020). This novel strategy takes advantage of cellular unique properties, such as circulating in the bloodstream for a period of time, abundant surface ligands, targeting (cancer) cells, flexible morphology, through challenging biological barriers as well as cellular signaling and metabolism, to maximize therapeutic outcomes as well as minimize side effects (Su et al., 2015). Cell-mediated DDS has become a new field of medicine, which enables the targeted delivery, prolongation of circulation time while reducing cellular and tissue toxicities. This system for drug delivery and targeted release represents a novel disease-fighting strategy for a range of human disorders.

In this review, different cell types used as carriers for various therapeutic agents are discussed, summarizing the existing designs for constructing cell-mediated DDS and providing perspectives on the future direction of ‘live’ drug delivery.

## 2. Cells used for cell-mediated drug delivery

The human body contains a variety of cells with different physiological functions, including long circulation in the blood, site-specific migration, and physical barriers crossing, etc. (Tan et al., 2015). Specifically, circulating cells serve as ideal drug delivery carriers due to their unique features, such as unparalleled systemic circulation, high fluidity, natural delivery mechanisms, and the ability to pass the bloodstream without immunogenicity. These characteristics are derived from the unique structure, mechanical properties, and surface ligands of each particular cell type. It is worthwhile to select certain types of cells to deliver drugs with retained cell structure and function.

In addition, the use of circulating cells as delivery vectors is beneficial because it can significantly reduce immune clearance and prolong the biological half-life of the delivered drug. Recently, the cell-based drug carriers have been emerging as a hot topic and attracted plenty of interests, including RBCs, Platelets, Stem Cells, Leukocytes, and immunological cells (Godfrin et al., 2012; Batrakova & Kabanov, 2013; Stuckey & Shah, 2014), whose properties are summarized in Table 1.

**Table 1.** Properties of RBCs, platelets, stem cells and leukocytes.

	RBCs	Platelets	Stem cells	Leukocytes
Amount (/mL blood)	4.2 to 6.0 × 10^6^	1.5 × 10^5^ to 4.5 × 10^5^	Unknown	4 × 10^9^ to 11 × 10^9^
Diameter (µm)	∼7	1–4	30–40	7–15
Location	Blood circulation	Megakaryocytes in the bone marrow	Circulation, tissues and organs	Circulation, tissues and organs
Life span	100–120 days	7–10 days	Depending on cell types	A few days; years for memory lymphocytes
Functions	Transporting oxygen	Hemostasis	Tissue repair and regeneration	Immune defense
Pros as DDS	Longest circulating half-life; large surface and volume; RES targeting	Extending their circulation time; Targeting capabilities; tumor cell-induced platelet aggregation	Easy harvesting and cultivation; tumor homing efficiency	Capable of cross biological membrane; tumor-homing; immune response

### 2.1. Red blood cells (RBCs)

Erythrocytes, red blood cells (RBCs) are the most abundant cells in the human blood, accounting for more than 99% of the total blood cells. About 2 million new RBCs are continuously produced per second in the human body (Muzykantov, 2010). Specifically, RBCs are non-nuclear biconcave disks with a thickness of about 2.5 μm and a large internal capacity volume of 185–191 μm (Su et al., 2015). In addition, the RBCs in the osmotic solution grant them a high surface area. This allows them to be reversible when navigating through capillaries that are typically smaller than the cell itself, thereby promoting red blood cells to be the most mobile circulatory cell. RBCs are also highly flexible, which further facilitates this navigation and reversible shape through the blood vessels.

Utilizing hemoglobin as an iron-containing protein, the major function of RBCs is to transport oxygen throughout the body and travel about 250 km through the cardiovascular system (Muzykantov, 2010). In addition to oxygen, RBCs can carry different valuable payloads, ranging from therapeutic agents to imaging contrast agents (Pierige et al., 2008). Since RBCs can limit the unintended extravasation and extend the time of systemic circulation, it has become an ideal drug delivery vehicle for extensive research.

RBC-based drug delivery has attracted increasing attention for a large number of reasons. RBCs can be easily separated, frozen, and stored for an extended period of at least 10 years (Ji et al., 2020). RBCs in the blood circulation can act as a reservoir for sustained drug release due to their long cycle times, availability, high biocompatibility, biodegradable, non–immunogenic as well as large surface and volume (Muzykantov, 2010). Besides, the biconcave shape and non-nuclear architecture allow RBCs to encapsulate many drugs. These make them a valuable systemic drug release platform (Kolesnikova et al., 2013).

However, there are still some challenges in using RBCs as drug delivery vectors. The RES system can quickly identify and eliminate modified RBCs before they reach the disease site, making non-RES targeting particularly challenging. Compared with natural ones, the carrier RBCs are more likely to be trapped in RES after injection due to the loss of natural integrity during drug loading (Laura et al., 2013; He et al., 2014). Drugs may also be released from destructed cells and result in cytotoxicity. In addition, RBCs are generally not as selective as other circulating cells in terms of targeting disease sites and promoting the healing process.

#### 2.1.1. Drug encapsulation in RBCs

**Figure 1.**
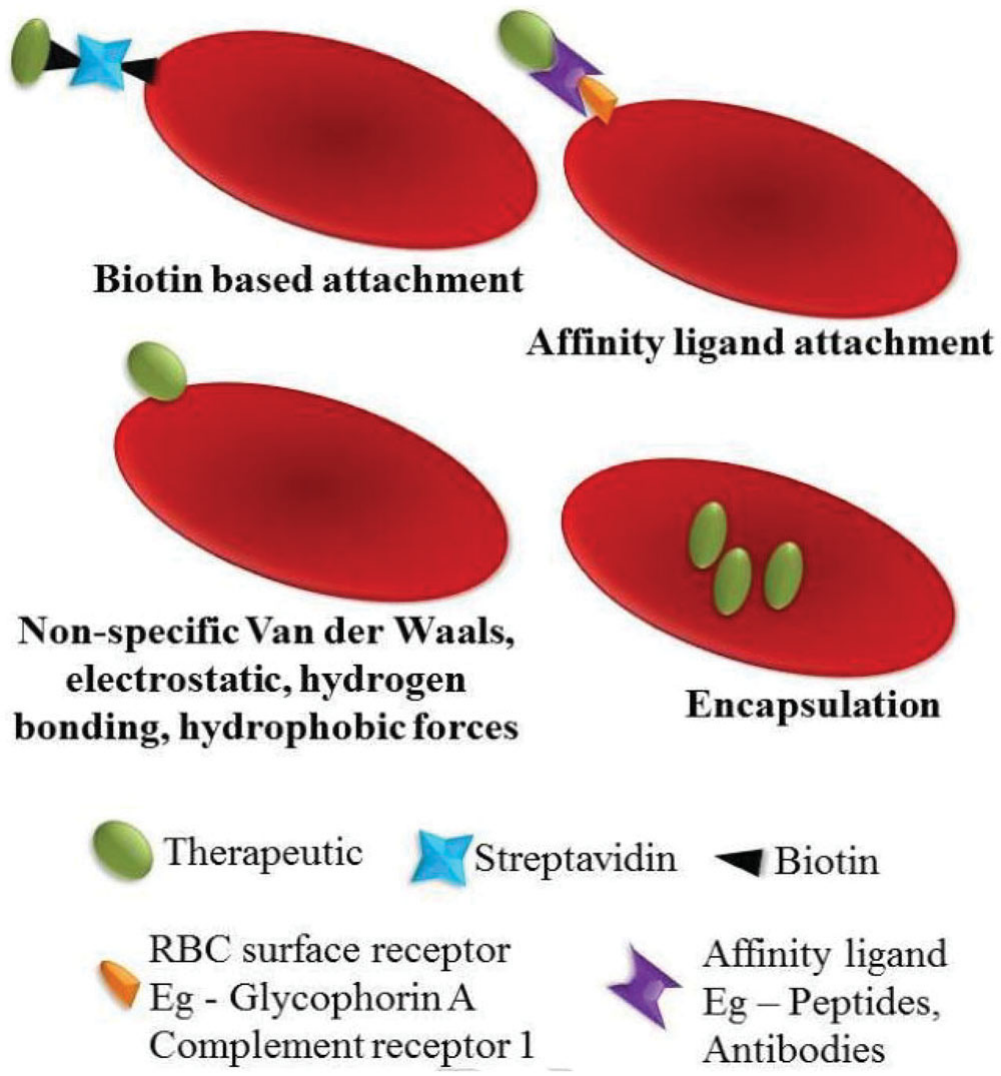
Strategies of RBCs loading. The therapeutic agents can be encapsulated into RBCs or attached to the surface of the RBCs by biotin, affinity ligands or nonspecific interactions, such as van der Waals forces, hydrogen bonds, electrostatic or hydrophobic forces. Reprinted with permission from Ma et al. (2020). Copyright ©2017, Elsevier B.V.

A large number of the desired drug molecules have been encapsulated into RBCs with the specific aim of using carrier RBCs as a slow vascular drug delivery system (Figure 1). The most frequently used methods include endocytosis of the drug (Favretto et al., 2013), electroporation (Nicolau, 2015), and osmosis-based method (Favretto et al., 2013) which are always followed by resealing and co-incubation that are commonly used for some membrane-active drugs (Hu et al., 2012). These techniques allow the drug to maintain its activity while increasing the loading efficiency from 10% to 77% (Favretto et al., 2013; Nicolau, 2015). However, the above methods still have some shortcomings.

In the case of loading agents into RBCs, most of the methods discussed above showed may cause the disruption of the cell membrane and result in a partial and irreversible deterioration of the structural integrity (He et al., 2014). The loss of hemoglobin and cytoskeleton through the pore is another fact that cannot be ignored, because the physiological function and structural integrity of RBCs may have been impaired. This may increase the chance of being recognized and cleared by the human body (He et al., 2014).

Recently, another new encapsulating method is developed to load proteins into RBCs is to use cell-penetrating peptides. The advantage of this method is that it would not lead to perturbation of the cell membrane and hence could maintain the functionality of the RBCs (He et al., 2014). He et al. showed that when L-asparaginase and protamine conjugates were encapsulated in RBCs, no hemoglobin escaped and RBCs retained their physical properties. Numerous other pharmacological agents have been successfully encapsulated into RBCs under *in vitro* and *in vivo* conditions and in a murine model of HIV infection (Alessandra, 2009).

#### 2.1.2. Drug attachment on RBCs surfaces

Over the years, RBCs have been used as diagnostic tools for *in vitro* agglutination research by the presence of external binding molecules. More recently, several methods have been developed to couple molecules using a variety of covalent and non-covalent crosslinkers of interest to RBCs for their *in vivo* use. By using the receptor-specific ligands and some binding agents (such as bio–bridge method) directly and indirectly, the therapeutic agents can be attached to the RBCs surface (Figure 1).

In general, the biological bridge includes a range of strategies for loading cargoes by physical or chemical means. The avidin-biotin bridge is a common method to conjugate bioactive agents on the surface of RBCs (Clafshenkel et al., 2016). This strategy has been used to attach various proteins RNA and DNA-based therapeutics to the membrane of RBCs (Rossi et al., 2019) to obtain recovery of yield with up to 90% but did not affect the cell’s 24 h survival in circulation (Murciano et al., 2003). Biopharmaceuticals, like HIV-1 TAT protein and bovine serum albumin, could all be conjugated to RBCs surfaces by the bio–bridge method.

Several antibodies and peptides have also been used as bridges to achieve targeting of antigens or immune antigens to the RBCs surface (Kontos et al., 2013). Studies have shown that ERY1 peptide (specifically binding to mouse blood glycoprotein-A) conjugated ovalbumin can bind directly and specifically to mouse RBCs *in vivo* after intravenous injection (Kontos et al., 2013).

In addition, nonspecific van der Waals forces, electrostatic, hydrogen bonds, and hydrophobic forces can also be used to attach therapeutic agents to RBCs surfaces (Chambers & Mitragotri, 2004). RBCs are used to deliver fibrinolytic agents because they can take advantage of the circulatory properties to reach the blood clots in the vascular endothelial structures without infiltrating into tissues (Ma, Gao, et al., 2020). Various therapies using RBCs as carriers are currently in clinical trials. However, the major limitation of using RBCs as drug carriers is that the loading of the RBCs would result in a change in its structural and functional properties, which in turn hinders *in vivo* compatibility.

### 2.2. Platelets

Platelets, cells produced from megakaryocytes in the bone marrow, are small, anucleate, discoid shaped blood cells that play a fundamental role in hemostasis (Parisa & Ashutosh, 2014). Under normal conditions, circulating platelets are presented at a concentration of 1.5 × 10^5^ to 4.5 × 10^5^ per µL of blood (Thon & Italiano, 2012). Platelets are the natural carriers of an enormous amount of biologically active proteins within their cytoplasmic granules that are critical to normal platelet function and are released upon platelet activation.

However, platelets still have several inherent properties that give rise to the interest of delivery systems. First, platelets have high storage and trafficking capacities, which allows them to contain many biologically active proteins within the cytoplasmic granules (Sun, Su, et al., 2017). Second, platelets can absorb nearby small molecules to protect them from immune surveillance (Sarkar et al., 2013), thereby extending their circulation time. Third, platelets have excellent targeting and release capabilities, and platelets naturally specific target the sites of injury, or sites of higher density of proliferating cells. In addition, platelets can be activated by tumor cells by tumor cell-induced platelet aggregation (Plantureux et al., 2020), which causes platelets to release their drug load around the tumor site. Thus, platelets deliver the loaded drug to the tumor cells, thereby reducing negative reactions in normal tissues.

**Figure 2.**
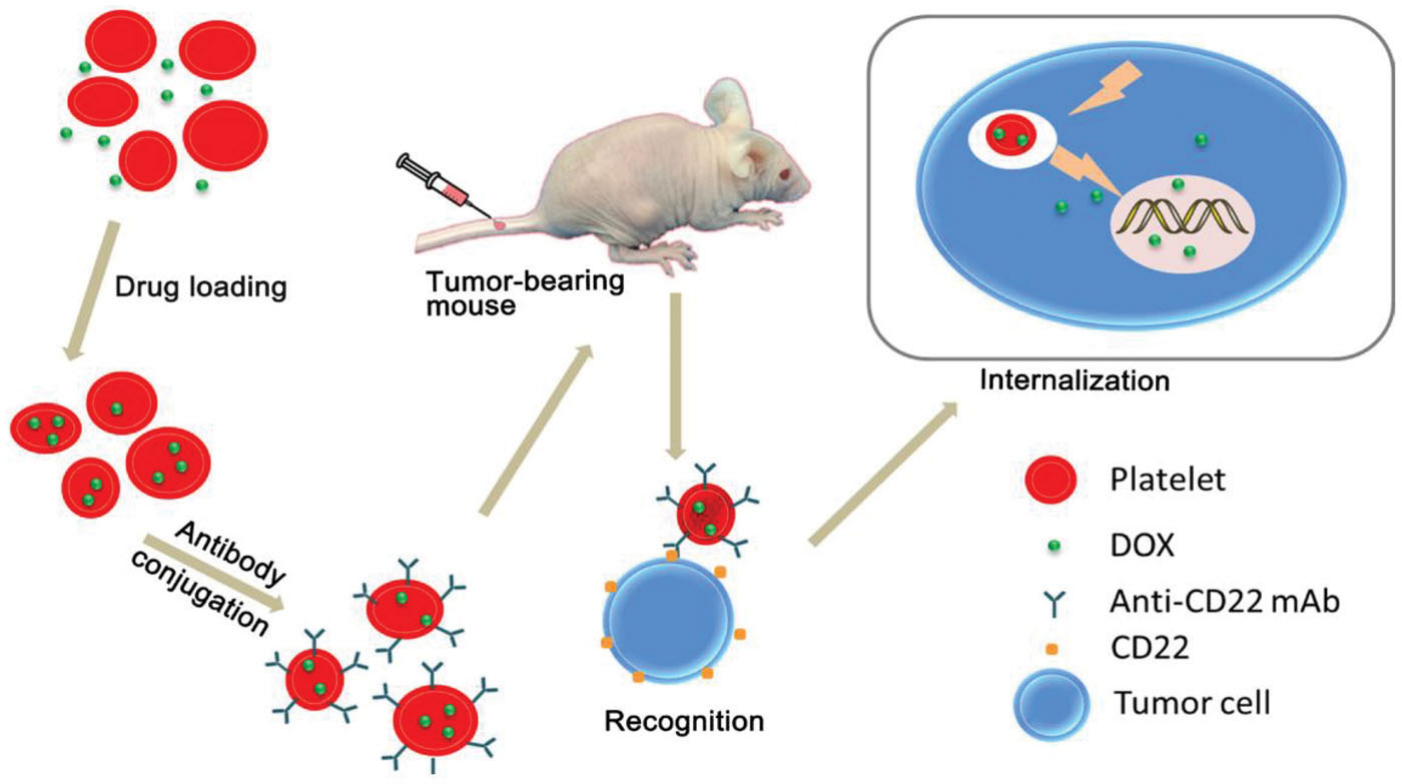
Schematic representation of the preparation of DOX-platelet-CD22 and its enhanced anti-tumor activity. DOX-platelet-CD22 can specifically target tumor cells by antigen-antibody binding and then internalize to exert cytotoxic effects. Reprinted with permission from Xu et al. (2017). Copyright ©2017.

When used for drug delivery, platelets can be loaded with therapeutic proteins and perform as circulating reservoirs. Studies have found that platelets are used to deliver coagulation factors by using their natural function in thrombogenesis at the site of vascular injury, allowing drugs to be delivered directly to the site of injury where they accumulate and release their contents (Shi et al., 2007). Rao et al. presented a platelet-facilitated photothermal tumor therapy (PLT-PTT) strategy. They found that the PTT therapy enhanced PLT-AuNRs targeting to the tumor sites, which in turn improved PTT effects by feedback, demonstrating the unique self-enhancement of PLT-PTT in cancer therapy (Rao et al., 2018). Doxorubicin hydrochloride, a potent anti-cancer drug, was loaded into platelets and resulted in significantly higher cytotoxicity to tumors both *in vitro* and *in vivo*, as compared with the free drug in Ehrlich ascites carcinoma (EAC) bearing mice (Sarkar et al., 2013). Similarly, there is a study reporting a new drug delivery system (DOX-platelet-CD22), which possesses several ideal qualities that minimize adverse effects and maximize therapeutic potential, including excellent biocompatibility, long circulation time, and precise targeting (Xu et al., 2017). Figure 2 shows a schematic of the mechanism of the drug delivery process.

### 2.3. Stem cells

Stem cells are self-renewable with the potential to differentiate into various cell types and therefore essential in tissue repair and regeneration. They are broadly classified into two categories according to their source and plasticity: embryonic stem cells and adult stem cells (Su et al., 2015). In particular, adult stem cells, such as hematopoietic stem cells and mesenchymal stem cells, can be obtained directly from patients, cultured and expanded *in vitro*, and used for subsequent autologous transplantation, which has attracted considerable interest in recent years (Corsten & Shah, 2008). Additionally, stem cells have the advantages of easy harvesting, easy cultivation *in vitro*, and can be induced to differentiate into specialized cells under certain conditions. These make stem cells promising candidates for targeted drug delivery (Su et al., 2015).

Current stem cell therapies including those for neurological disease, heart disease, and cancer can all be improved by incorporating therapeutics into stem cells. Although the main focus of stem cell therapy is cell replacement or tissue regeneration, their ability to migrate to tumor tissues also makes them an ideal delivery vehicle for cancer therapy (Corsten & Shah, 2008). Tumor homing mediated by inflammatory factors, tumor-derived factors, and tumor-specific receptors is actually the essence of stem cells (Reagan & Kaplan, 2011). Normally, it takes 2–4 days for stem cells to migrate to the tumor site (Aboody et al., 2000). Many types of stem cells, including mesenchymal stem cells and neural stem cells, have been shown to have the ability to migrate to the tumor microenvironment, and are therefore widely used for tumor-specific drug delivery (Stuckey & Shah, 2014). For example, the delivery of the oncolytic drug 5-fluorouracil by neural stem cells resulted in a reduction of approximately 80% of tumor masses in intracranial gliomas within 2 weeks (Aboody et al., 2000). In another study, MSC modified with Dox-loaded silica nanoparticles also successfully tracked glioma cells and released Dox to kill cancer cells (Linlin et al., 2011).

The use of stem cells is not limited to tumor targeting. Stem cells also exhibit therapeutic effects on a variety of other lesions as well as neurodegenerative regions, thus representing a unique delivery vehicle with a broad therapeutic range (Porada & Almeida-Porada, 2010).

### 2.4. Leukocytes

Leukocytes, a type of white blood cells that form a part of the innate and adaptive immune system and respond, are involved in protecting the body against infectious diseases, cleaning cellular debris and foreign invaders (Gajewski et al., 2013; Mitchell & King, 2015). Normal human blood contains 4 to 11 × 10^9^ leukocytes per mL (Mitchell & King, 2015). According to their physical and functional characteristics, the five existing leukocytes can be divided into five categories, including neutrophils (40–75%), lymphocytes (20–45%), monocytes (2–10%), eosinophils (1–6%) and basophils (less than 1%). Table 2 lists three major types of leukocytes in human, and their basic properties (Msom, 2020).

**Table 2.** Properties of neutrophils, and lymphocytes and monocytes/macrophage.

	Amount in human blood	Diameter (µm)	Lifespan
Neutrophil	50–70%	10–12	3–4 days
Lymphocytes	20–35%	6–12	B cells: 4 days up to 5 weeks T cell: month to years
Monocytes/macrophage	2–8%	∼25	10–20 h

Compared with other blood cells, leukocytes possess the following peculiarities. The unique features of leukocytes, such as their ability to travel to a specific site of disease as well as to transmigrate through biological barriers into non-vascular areas (Muller, 2003). Leukocytes can interact with cancer cells both in the bloodstream and at the site of solid tumors and provide unique opportunities for delivery to areas that are otherwise difficult or impossible to reach by traditional drug delivery approaches. Further, leukocytes and tumor cells share similarities in physical properties and adhesive properties (Cheung et al., 2011). Besides, the ability of leukocytes to genetically homing to the inflammatory/tumor regions makes it a promising carrier candidate for targeting the delivery of chemotherapeutic agents and tumor microenvironmental modulators (Turley et al., 2015).

A suitable strategy for leukocyte-derived drug delivery is to take advantage of the biocompatibility and biological function of living leukocytes to prolong the life of drugs *in vivo* and to target inflammatory tissue with leukocytes for site-specific drug delivery (Huang et al., 2018). The loading cargoes by leukocytes are also well-investigated and summarized, mainly including membrane bonding and encapsulation. To achieve cell surface functionalization, therapeutic agents bind to leukocytes membranes primarily through three techniques, including receptor-mediated adhesion, covalent binding, and selectin-mediated adhesion (Doshi et al., 2011). Another approach is to encapsulate the therapeutic agent inside the leukocytes. For leukocytes, a hypotonic/reseal method can be used to load the therapeutic drugs. Leukocytes^,^ encapsulation conditions (e.g. osmotic pressure and dialysis reduction) are slightly altered compared to encapsulation by red blood cells (Sungmun, 2014). Both small molecule and macromolecular therapeutics can be encapsulated by leukocytes by passive diffusion.

Although the lifespan of leukocytes (up to 20 days) is generally shorter than the lifespan of RBCs, their special functions make them attractive as drug delivery vehicles because leukocytes are involved in various immune responses, cellular interactions, and cell adhesion (Su et al., 2015). Therefore, Neutrophils, monocytes/macrophages, and lymphocytes are all potential candidates for delivering therapeutics to treat various diseases.

#### 2.4.1. Neutrophils

Neutrophils, also known as polymorphonuclear granulocytes (PMNs) that contain distinctive cytoplasmic granules, are the most abundant immune cell population in the human peripheral blood and are generated from the bone marrow (Coffelt et al., 2016).

**Figure 3.**
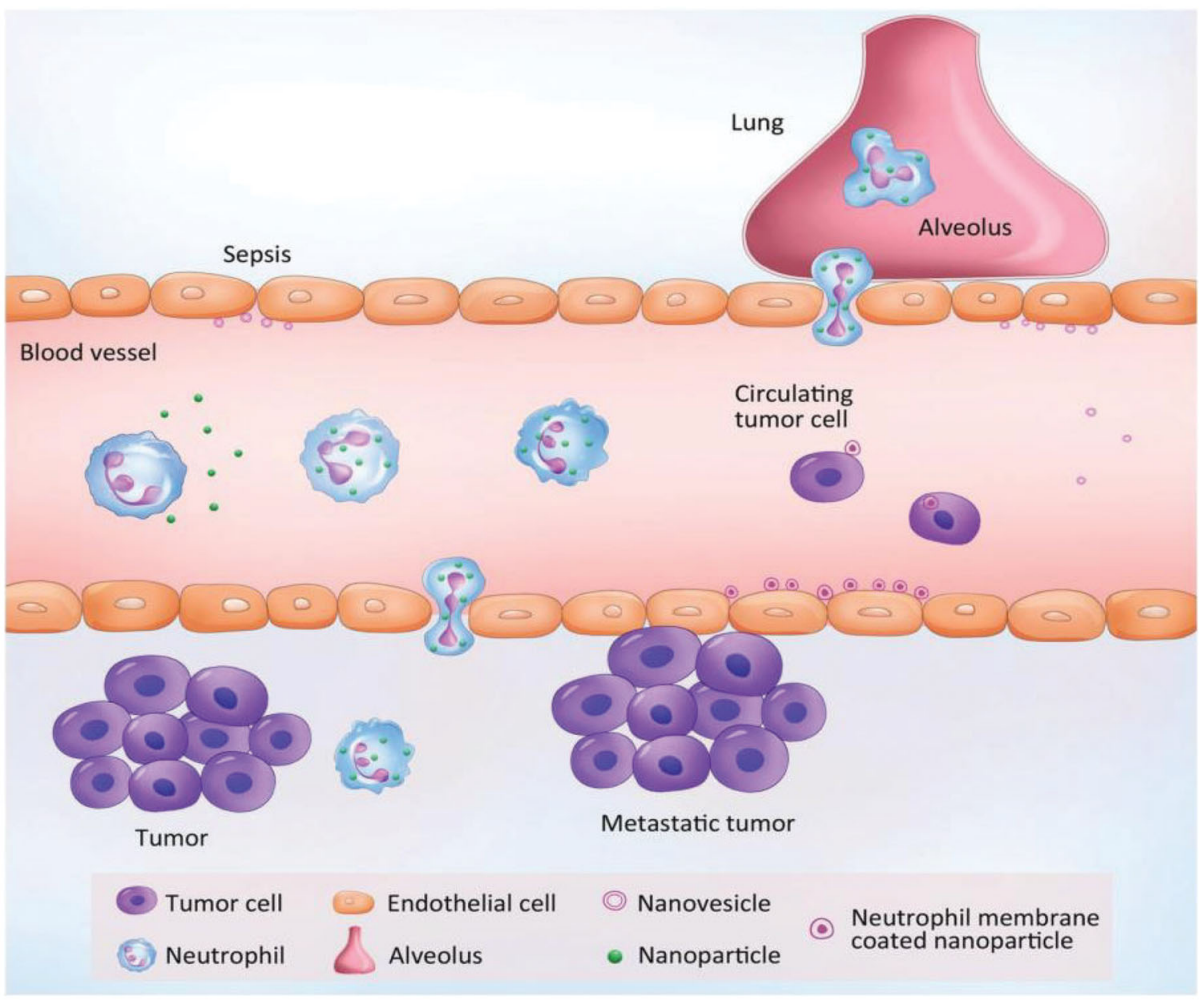
Neutrophil-mediated targeted delivery of nanotherapeutic drugs and neutrophil membrane-derived vesicles for targeted drug delivery. Reprinted with permission from Chu et al. (2018). Copyright ©2018, Drug Delivery.

More than 2 × 10^11^ neutrophils may be produced per day and represent 50–70% of leukocytes (Dong et al., 2017). Unlike other WBCs, neutrophils possess the natural chemotaxis to inflammatory signals (Chu et al., 2018). They are not limited to specific circulation areas and can move freely through the walls of veins and body tissues to instantly attack antigens. Here, we discuss the status of a neutrophil-based drug delivery system (summarized in Figure 3).

There are several novel properties that make neutrophils potential carriers to deliver nanotherapeutics: (1) Neutrophils are the first cell type to arrive at the sites of infection or inflammation, producing cytokines to recruit other cells, and are cleared after a few days (Amulic et al., 2012). (2) while the lifetime of neutrophils is short in circulation, the number of neutrophils are the most abundant cells during acute inflammation compared to monocytes/macrophages, which would quickly increase the drug delivery (Kolaczkowska & Kubes, 2013). (3) as a response to inflammation, the number of neutrophils can be increased by a hundredfold or more in a short period of time. Therefore, targeting of neutrophils can improve therapeutic efficacy and can be transformed (Dong et al., 2017).

**Figure 4.**
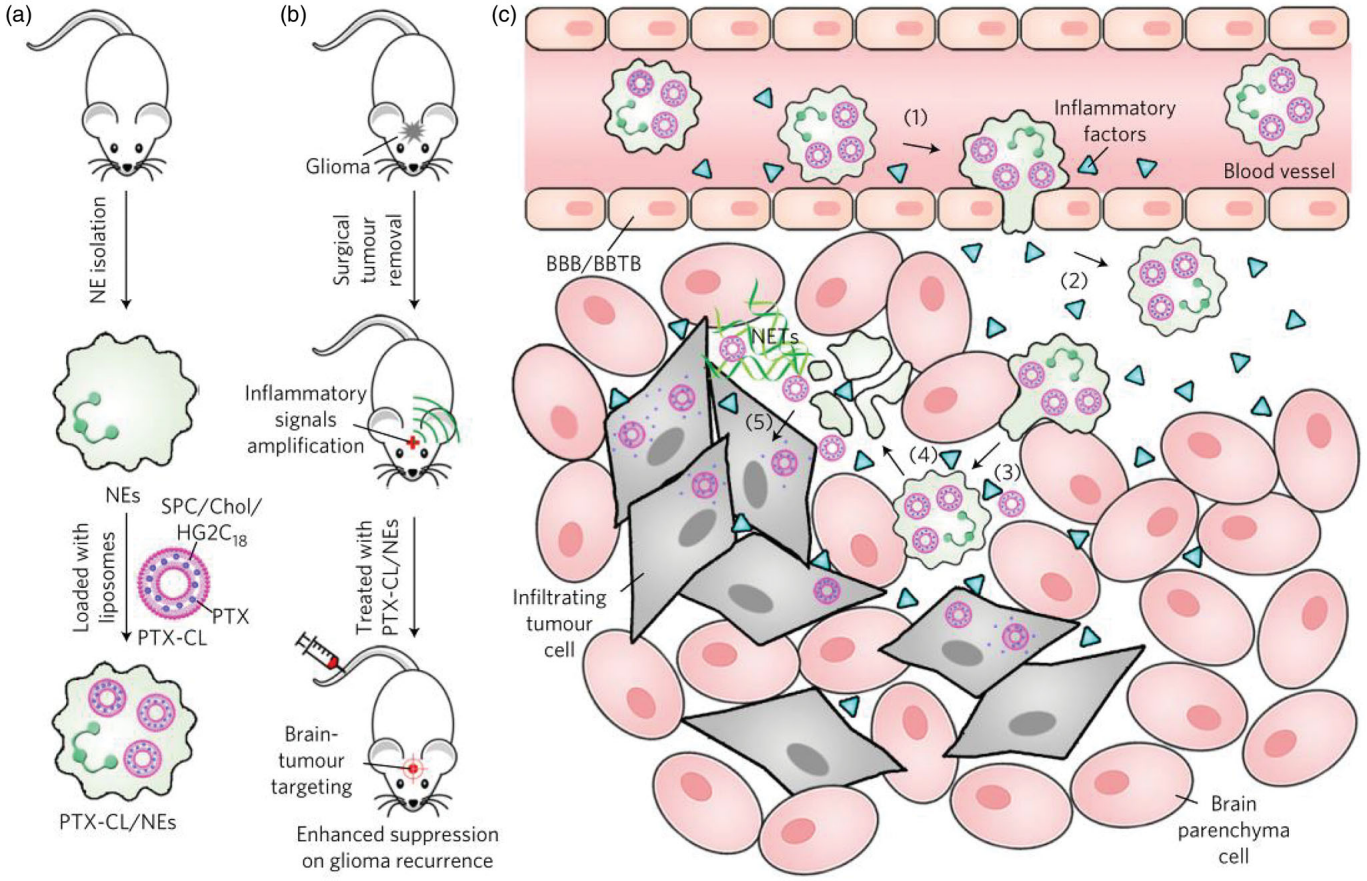
Schematic representation of NE-mediated anticancer drug delivery designed to inhibit postoperative glioma recurrence. (a) Schematic diagram of the preparation of PTX-CL/NE. SPC, soybean phosphatidylcholine; Chol, cholesterol; HG2C_18_, 1,5-dioctadecyl-N-histidyl-L-glutamic acid. (b) Schematic showing how PTX-CL/NE inhibits recurrence of postoperative glioma in mice. Surgical resection of the tumor expands the inflammatory signal in the brain, which allows PTX-CL/NE to target brain tumors, release PTX and inhibit glioma recurrence. (c) Schematic showing how PTX-CL/NEs can target glioma after intravenous injection of brain tumors have been surgically removed: (1) inflammatory factors direct the movement of PTX-CL/NE along the chemotaxis gradient; (2) PTX-CL/NE is transferred to the inflamed brain via BBB/BBTB; (3) PTX-CL/NEs penetrate the infiltrating tumor cells; (4) PTX-CL/NE is overactivated by concentrated cytokines and releases NETs, resulting in concomitant release of PTX-CL; (5) PTX-CL delivers PTX to infiltrating tumor cells to produce an anti-tumor effect. NET, which consists mainly of DNA from NE, is a fibrous extracellular matrix that is released by NEs and is overactivated by inflammatory cytokines. Reprinted with permission from Xue et al. (2017). Copyright ©2017, Macmillan Publishers Limited, part of Springer Nature. All rights reserved.

The short life span of neutrophils limits their use in DDS, but their ability to migrate and transport immediately makes them attractive as drug carrier candidates. For example, Xue et al. (Xue et al., 2017) successfully prepared PTX-CL-loaded neutrophils by using neutrophils to deliver carriers of nanoparticulated chemotherapeutic agents, aiming to inhibit the recurrence of glioblastoma by surgically removing tumors (Figure 4). They found that the PTX-CL-loaded neutrophils showed a chemotactic response under inflammatory stimulation, and they actively generated superoxide and burst to release their PTX-CL cargo once inside the inflammatory region. In addition, the role of neutrophils is critical in cancer because it plays a positive role in the progression of cancer. Studies have shown that chemotherapeutic drug-loaded murine neutrophils in response to inflammatory signals have potent tumor targeting and superior biological safety in mice (Xue et al., 2017). Recently, Ju et al. developed a neoadjuvant therapy based on human neutrophils cell drugs for effective gastric cancer therapy. Abraxane/Neutrophils can accumulate and over-activate at the tumor site, forming neutrophil extracellular traps (NETs). At the same time, Abraxane is released that can be internalized by neighboring tumor cells, resulting in strong anti-tumor efficiency (Ju et al., 2019). Neutrophils are capable of trans-endothelial transfer through the vascular barrier into tissues for rapid response to injury and infection. Thus, neutrophils can be an excellent vector that mediates the transport of nanoparticles across the vascular barrier. Chu et al. (2015) proposed a new method for transporting nanotherapeutic drugs to deep tissues through the neutrophil transmigration pathway. Overall, these findings reveal a new strategy for designing therapeutics which are capable of in situ hitchhiking neutrophils for targeted drug delivery.

#### 2.4.2. Lymphocytes

Lymphocytes, the second most abundant human leukocytes of humans, are identified by their large nucleus surrounded by a thin layer of cytoplasm (Fliervoet & Mastrobattista, 2016). The average diameter of lymphocytes is 7–15 µm. Lymphocytes are mainly found in the circulation and central lymphoid organs such as the spleen, tonsils, and lymph nodes (Gel’m et al., 2019). There are three main subtypes of lymphocytes, including T-cells, B-cells, and natural killer cells. Among them, T cells and B cells are the major types responsible for the adaptive immune system.

T cells are primarily responsible for cell-mediated immunity and can be broadly divided into helper T cells, cytotoxic T cells, and memory T cells according to the different functions. When an antigen in the body appears, the antigen-presenting cells would recognize and present the antigen to T cells. Then, helper T cells stimulate toxic T cells and subsequently kill abnormal cells (Shen et al., 2020). Memory T cells retain antigenic information in order to generate a rapid immune response to previously encountered antigens (Clarke et al., 2019). B cells are produced in the bone marrow and involved in humoral antibody-secretive adaptive immunity. On the other hand, B cells synthesize antibodies and antigens to promote the production of memory B cells (Mauri & Bosma, 2012).

**Figure 5.**
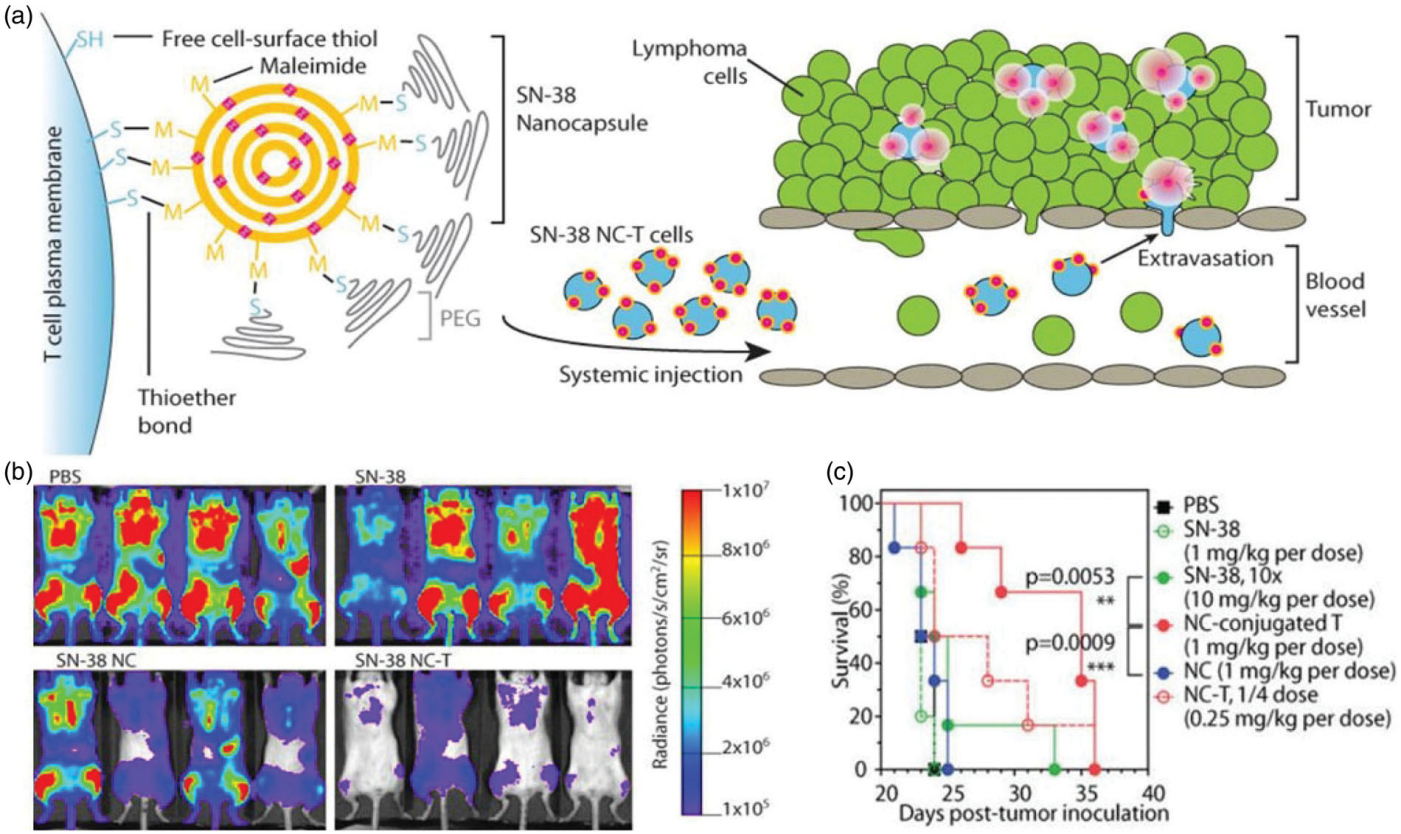
(a) T cell functionalization and cell-mediated delivery of SN-38 nanocapsules (NC) to tumors. (b) Bioluminescence images of tumor burden on day 16 of mice treated with PBS, SN-38, SN-38 nanocapsules and SN-38 nanocapsules were sputum on T cell vector (NC-T). (c) Overall survival. ***p < .001 by log-rank test. Reprinted with permission from Huang et al. (2015). Copyright ©2015. American Association for the Advancement of Science.

T lymphocytes have three main functions which make them important in cancer immunotherapy: destroying foreign invaders directly, activating nearby immune cells through cytokine secretion, and expanding B cell response. One technique is used to isolate T cells from the patient’s blood, which are then conjugated with a chimeric antigen receptor (CAR) on the surface of T-cells (Zhang et al., 2014; Dai et al., 2016). The modified T cells are able to home in the tumor site because the expression of the targeting antigens on the T cell directs their tumor to homing. However, the main obstacle to cell-based therapies is the rapid decline in the viability and function of transplanted cells. To optimize these cell therapies, Stephan et al. (Zhang et al., 2014) have presented a new strategy for attaching drug-loaded nanoparticles to the surface of transplanted T cells. Irvine’s group also proposed a strategy to utilize polyclonal T cells as carriers to deliver nanotherapeutics into lymphomas which are hematological cancer (Huang et al., 2015). In the study, they generated a complex delivery system of T cells backpacking lipid nanocapsules (NCs) and SN-38, which was loaded in NCs. This T cell-mediated delivery reduced tumor growth significantly and increased survival (Figure 5).

In addition to the strategy of backpacking nanoparticles to the cell surfaces, loading nanoparticles within T cells may be another option. Doxorubicin conjugated magnetic nanoparticles (Target MAG-Dox) (Steinfeld et al., 2006) were used and the properties of nanoparticle-laden T cells were analyzed *in vitro*. Clearly, lymphocytes can serve as a potential platform for the specific delivery of drugs.

#### 2.4.3. Monocytes and macrophages

Monocytes, kidney-shaped nuclei, and clear cytoplasm are leukocytes that are the precursors to macrophages (Zargar et al., 2019). They are produced from stem cell precursors in the bone marrow. Monocytes are typically stored in the spleen and circulate in the bloodstream until terminally differentiating into macrophages in response to various stimulations. Macrophages are characterized by their large size (5–50 µm in diameter), irregular shape, remarkable immunological versatility (Ciaccia, 2011). Typically, circulating macrophages have a half-life of 1–3 days.

Macrophages are vital regulators of the innate immune system (Mitchell & King, 2015). Many efforts have been taken to develop macrophages for drug delivery due to their unique features that are attractive for drug delivery. First, the typical feature of macrophages is their ability to migrate along a chemoattractant gradient to pathological regions, usually sites of inflammation and tumors (Dong et al., 2017). Second, macrophages can recognize and effectively clear foreign materials such as polysaccharides, endotoxins, and low-density lipoproteins traveling in the blood (Pierige et al., 2008), providing an opportunity to load nanoparticles into macrophages. Third, macrophages can target a variety of pathological conditions, including cancer, infectious, and autoimmune diseases (Parisa & Ashutosh, 2014). In addition, macrophages also tend to localize in hypoxic regions. Therefore, it is desirable to target anti-cancer drugs to the areas of hypoxic tumors that are often associated with poor drug delivery. Fourth, macrophages are activated by a variety of stimuli at the site of the disease, which can trigger the release of intracellular contents including drugs. As a result, macrophages are attractive as carriers for therapeutic delivery.

Owing to intracellular degradation, direct loading of free drugs into macrophages would probably lead to low drug loading, premature drug inactivation, and limited drug delivery to target cells (Ma, Cao, et al., 2020). Nanoparticles may be an ideal carrier for cell-mediated drug delivery since the incorporation of drugs into nanoparticles will help reduce drug disintegration in monocytes/macrophages. Choi et al. (Choi et al., 2007) successfully prepared a nanosized gold shell and allowed it to be engulfed by macrophages *in vitro*. Such nanoparticle-incorporated macrophage exhibited enhanced infiltration into tumor spheroid and induced tumor necrosis upon near-infrared radiation. Another study demonstrated that gold nanorods loaded into RAW264.7 macrophages when injected intratumorally in the tumor core could result in 95% tumor suppression on day 14 with no tumor recurrence (Li et al., 2016). Studies have reported a strategy to create ‘backpacks’ of nanoparticles onto the surface of macrophages to avoid problems that stem from the internalization of therapeutics into macrophages (Doshi et al., 2011). However, at present, achieving long-term fixation of macrophage surfaces is challenging since it is necessary to precisely adjust the shape and mechanical properties of these nano-scale backpacks.

## 3. Encapsulation/conjugation of drugs/particles to cells

Cells, being biological entities, are comprised of biomolecules such as proteins, lipids, and polysaccharides which offer a range of functional groups and surface properties that drugs or drug-loaded particles can attach onto or internalized into cells to form drug-cell or particle-cell complexes to obtain cell-mediated drug delivery. The basic requirements for drugs and drug-loaded particles are nontoxic to cell carriers, low toxicity, degradability, and controlled release. For drug/particle-cell complexes, it is required to have a strong binding between payloads and cell carriers, long circulation time, and low immunogenicity. Both non-covalent and covalent techniques have been used to attach or conjugate nanoparticles to the surface of cells (Stephan & Irvine 2011). Each method has unique advantages and disadvantages and should be considered based on the cells and nanoparticles used for cell hitchhiking. To date, drug vehicles can be added to cells via (i) ligand-receptor attachment, (ii) electroporation, and (iii) covalent coupling.

### 3.1. Ligand-receptor attachment

Ligand-receptor interaction, one of the most basic methods for cells to communicate with the environment, is a method that does not require cell modification mediated by receptors that naturally occur on the surface of overlapping cells. Cell receptors are generally embedded in the plasma membrane surface, and the plasma membrane surface contains specific sites for ligand binding. The advantage of ligand-receptor attachment is that attachment to target cells is: (i) reliable and reproducible, (ii) purified target cells are simpler, (iii) the selectivity and specificity of ligand-receptor attachment can be exploited by active nanoparticle targeting strategies, and (iv) in the case of specific ligand-receptors can be converted into *in vivo* pairs. Therefore, this method can also be used to load particles onto cells. Given the relatively rich variety of receptors on the cell surface, various drug carriers can be connected to them through ligand-receptor interactions.

The current CD44-hyaluronic acid interaction has been used in a variety of cell types, mainly to mediate the attachment of cell backpacks to various leukocyte surfaces (Vasconcellos et al., 2010; Doshi et al., 2011). Most recently, HA derivatives and biocompatible thermosensitive polyethylene glycol methyl methacrylate form nanogels by the self-assembly method. After intravenous injection of nanogels, mice can be quickly engulfed by circulating macrophages within 13 min and stored in the liver and spleen. The study also showed that HA-CD44 binding can effectively attach nanocarriers to macrophages (Fernandes Stefanello et al., 2014).

**Figure 6.**
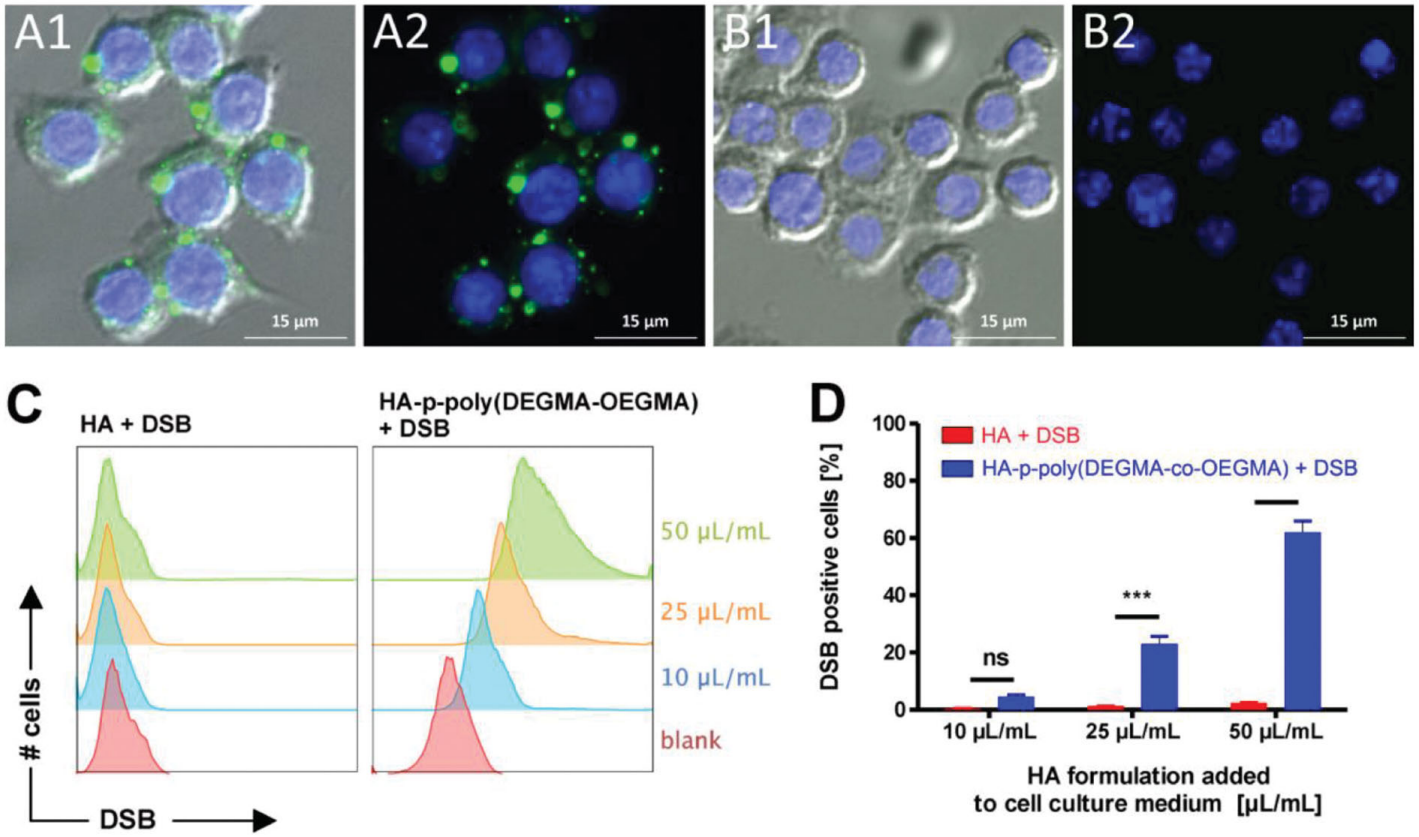
(A, B) Fluorescence microscopy images of RAW264.7 macrophages incubated with DSB loaded HA-p-poly(DEGMA-co-OEGMA) nanogels (A1, A2) and DSB with native HA (B1, B2). Cell nuclei are stained blue with DAPI and DSB is visualized in the green fluorescence channel. The right panels show only the fluorescence channels. The left panel depicts an additional overlay with the DIC channel. Bottom: (C) flow cytometry histograms recorded from RAW264.7 macrophages that were incubated for 1 h with different amounts of the respective HA/DSB formulations (i.e. with DSB loaded HA-p-poly(DEGMA-co-OEGMA) nanogels and DBS in the presence of native HA) per mL cell culture medium; (D) corresponding graphs depicting the percentage of RAW264.7 macrophages that were found positive for DSB after 1 h incubation with the respective HA/DSB formulations (*n* = 3, ***p < .001). Reprinted with permission from Fernandes Stefanello et al. (2014). Copyright © 2014 Acta Materialia Inc. Published by Elsevier Ltd.

Therefore, it is expected that this macrophage/nanogel complex can be used for the treatment of liver and spleen macrophage-related diseases (Figure 6).

The cell-particle encapsulation/conjugation also can be achieved by antibody–antigen interactions. Without any cell pretreatment, the silica nanorattles-doxorubicin drug delivery system can be effectively anchored to mesenchymal stem cells (MSC) through specific antibody–antigen recognition on the cell membrane interface (Linlin et al., 2011). After antibody–antigen recognition, each MSC cell can take up to 1500 nanorattles with high cell viability and tumorigenic ability, and the retention time in the cell is not less than 48 h, which is enough for the MSC to migrate to the tumor site (Linlin et al., 2011). This strategy may be developed as a reliable and scalable method for targeted tumor therapy with high efficiency and low system toxicity.

In addition, some receptors are only present in certain cell types, at which time cells can selectively absorb circulating particles without assembling cell-drug complexes *in vitro*. For instance, Ovalbumin, an antigen that binds to ERY1, recognizes RBC spontaneously within 30 min and remains tightly bound for at least 72 h after intravenous injection in mice (Kontos et al., 2013). However, to date, no drug-loaded particles have been developed to bound to cells through the specific interactions described above.

Therefore, ligand-receptor binding is an effective method for attaching particles to circulating cells. The discovery of new surface markers and advances in ligand-associated cell engineering are beneficial for ligand-based drug carrier design. Improving the efficiency of binding to specific cell types remains a challenge. It is also desirable to complete specific binding *in vivo* within the blood circulation.

### 3.2. Electroporation

Electroporation is a physical method that uses electrical pulses to form temporary holes on the surface of a cell under a strong external electric field (Tan et al., 2015). Due to the action of a high-intensity external electric field, cells are generally suspended in a conductive solution. The disorder of the phospholipid bilayer can cause temporary dysfunction of the cell membrane, resulting in the encapsulation of foreign molecules by each cell. In this process, whether the engulfed molecule can successfully enter the cell depends on the size of the molecule. If the engulfed molecule is larger than the pore size, the cell will maintain its shape. Conversely, the cells may swell and the cell membrane may be ruptured (Su et al., 2015). Studies have reported that various compounds (such as enzymes, nucleic acids, and anionic drugs like diclofenac sodium) have been encapsulated in red blood cells with sustained-release functions (Phua et al., 2014).

The messenger RNA was successfully loaded into whole blood cells by electroporation to develop an anti-tumor vaccine. Under the effect of cluster formation and externalization of the ‘eat-me’ signal of phosphatidylserine (PS) on the cell surface, the cell vaccine can be quickly distributed to the liver and spleen since it does not require additional pretreatment to achieve opsinization (Phua et al., 2014). This will cause the RES system to engulf and greatly shorten the blood circulation time. However, electroporation also has certain disadvantages. This method may damage the cell membrane and cause partially irreversible deterioration of the structural integrity, the recovery rate is usually very low (He et al., 2014). Therefore, it is necessary to optimize the applied voltage to maintain cell integrity in electroporation.

### 3.3. Covalent coupling

The cell surface is rich in proteins, and these proteins provide functional groups in the form of amines and thiols on different cell surfaces. These functional groups can be bioconjugated with molecules or particles. The available functional groups have the advantages of easy labeling and low reaction toxicity. Primary amines and mercapto groups are mainly used for covalent reactions. In the research, N-hydroxysuccinimide (NHS) ester group is used as the cross-linking agent to modify the primary amine by carbodiimide reaction. Current research uses methods to modify particles and cells to provide covalent bonds, which are stronger than adsorption or ligand-induced binding.

The sulfhydryl group in the cysteine-containing membrane protein can be coupled through maleimide-sulfhydryl conjugation. The maleimide group can be covalently conjugated to the surface of thiol-rich T cells by reacting to the maleimide group on the liposome. Using this strategy, each cell can bind up to 100 nanoparticles without causing toxicity or affecting cell function (Stephan et al., 2010). In addition, maleimide-functionalized liposome-coated NPs were conjugated on the surface of lymphocytes and MSCs. The attachment of NPs will not activate lymphocytes or alter stem cell tumor homing ability.

The covalent coupling has a stronger binding force than ligand-receptor interaction, which can prevent the drug from detaching during cell migration. A variety of conjugated chemical techniques can perform exclusive covalent reactions with amino or thiol groups naturally expressed on cells. These covalent combinations help to anchor the drug carrier to the cell surface, can circumvent the possible harmful loading technology, bypass the drug release problem, and have advantages over the phagocytic cells in terms of cell safety and drug protection. In addition, coupled drugs no longer encounter diffusion limitations.

## 4. Targeting strategies of cell-mediated DDS

As long as the drug carrier and circulating cells are assembled together *in vitro* or *in vivo*, the complex can be smoothly spread and diffused in the blood circulation and further transported to the desired target. The inherent ability of the carrier cells can achieve the controlled delivery of drugs/particles to specific disease sites. In this section, we will review the existing circulating cell-based targeting strategies for various goals.

### 4.1. Live cell-mediated targeting strategies

There are various natural targeting processes within the human body. Many circulating cells have the ability to target diseases such as cancer, wounds, and ischemic tissues. For example, as a first-line defender, leukocytes can quickly migrate into infected tissues with lipogenic pathogens. In addition, tumor homing is also one of the targeting mechanisms of living cells. These have stimulated researchers’ interest in targeting strategies using natural or genetically engineered cells as specific sites. Although cell-based targeting is still in the early stages of development, it has advantages such as high specificity and versatility compared to traditional strategies. Depending on the nature of the therapeutic agent and the intended applications, the therapeutic agent can be coupled to the cell surface or encapsulated within the cell.

A large number of cells, blood vessels, and interstitial tumor microenvironments are other important targets for therapeutic delivery mediated by circulating cells. Due to the lack of blood vessels in solid tumors, hypoxic cells are prone to resistance to traditional drug delivery methods. There are often monocytes, tumor-associated macrophages, and tumor-infiltrating lymphocytes in the tumor microenvironment, which indicates that white blood cells can enter deep into the tumor. Cui et al. found that monocytes/macrophages can be used as ‘Trojan horses’ to deliver gold nanoshells to low-oxygen areas of cancer, and the results show that free nanoshells cannot access these areas (Choi et al., 2007). Another study reported the development of dual-size/charge-switchable Trojan-Horse nanoparticles (pPhD NP) to achieve multimodal imaging-guided tumor eradication. In the tumor microenvironment, pPhD NPs are responsively converted into complete API nanothermology, thereby greatly promoting tumor penetration and cell internalization (Xue et al., 2018). This convertible Trojan-Horse horse nano platform has powerful delivery efficiency and versatile diagnostic functions, showing great potential for improving cancer treatment.

Cancer cells can be fatal when they reach a metastatic state. Unfortunately, due to the lack of blood circulation of metastatic circulating tumor cells (CTC), it has been a major challenge in recent years. CTC can automatically leave the primary tumor and sow metastases in the distance. More interestingly, studies have shown that tumor cells interact with leukocytes and endothelial cells through adhesion molecules, which provides a platform for targeting CTCs through leukocytes. Inspired by the rolling of selectin-dependent blood vessels, the study describes a method for targeting leukocytes in blood by directly targeting the apoptosis-inducing ligand TRAIL and the adhesion receptor E-selectin in the blood under shear flow, thereby effectively killing tumors (Mitchell et al., 2014).

Therefore, circulating cells have shown promise as a powerful drug delivery system with the inherent ability to detect diseases, cross biological barriers, and release drugs to disease sites. Compared to traditional drug delivery and nanomedicine, the natural ability to utilize different cells is a completely different targeting mechanism, where live cells act as a guide and driver for the delivery of therapeutic agents and NPs. This strategy also opens up new ways for the treatment of diseases that are difficult to achieve with traditional DDS.

### 4.2. Engineered targeting/binding

An engineered non-natural targeting/binding strategy has been developed for live cell-mediated DDS to maximize targeting efficiency, thereby allowing more flexibility and diversity in the binding pathway. Through this most popular method of antibody modification, cell carriers can automatically search for specific antigens and even trigger the removal of certain cells.

One research demonstrates that using the lipophilic ligand painting strategy, Lipophilic anti-CD45 and anti-CD20 (Rituximab) surface painted RBCs could be in one step converted into targeted entities that efficiently and specifically bind to CD45 positive leukocytes and CD19+/CD20+/CD45+ human lymphoma cells *in vitro* and *in vivo*, inducing therapeutic effect in the mouse model of lymphoma (Mukthavaram et al., 2014). The lipophilic ligand-coated RBC is a new tool that can be used to target blood-derived cells for experimental immunology and drug delivery applications.

Another method is to use an external magnetic field to enhance the accumulation of cellular carriers in the target. Tumor targeting with magnetic carriers induced by an applied external magnetic field (MF) relies on physical forces to enhance tumor accumulation of therapeutic agents. Magnetic nanomaterials can enable remote control of the location of drug vehicles including circulating cells by an external magnetic field and therefore could be applicable to a wide range of solid tumors.

SPION NPs have been widely explored due to their biocompatibility, ease of handling, and good MRI contrast. The surface of the IONPs outside the RBCs was further coated with polyethylene glycol (PEG), obtaining a DOX@RBC-IONP-Ce6-PEG structure with a long blood-circulation time and strong responses to an external MF. Both *in vivo* and ex vivo imaging data evidenced the highly efficient MF-induced tumor homing of those RBCs, further reducing the RES aggregation (Wang et al., 2014). This study demonstrates an RBC-based drug-delivery system with the assistance of MF-enhanced tumor-targeting could serve as a multifunctional platform that is safe and holds promise for imaging-guided combination therapy of cancer.

The combination of molecular binding and cell-mediated drug delivery is a new and emerging field. The techniques in cellular engineering and biomaterial modifications offer significant opportunities for targeted drug and nanoparticle delivery.

## 5. Conclusions and future prospects

There are many potential biomedical applications of cell-based drug delivery systems that open up new perspectives on the possibility of using our cells for therapeutic purposes. Compared with traditional targeted drug delivery and nano-drug strategies, cell-mediated drug delivery must consider additional factors. Drugs/particles must not be toxic to the cell carrier and should prevent cell changes after the drug is loaded (Liang et al., 2018; Deng et al., 2019). Therefore, more emphasis has been placed on anchoring therapeutic agents on the surface of living cells. Secondly, a reasonable selection of suitable cells is necessary to achieve high targeting efficiency of new cell-mediated drug delivery systems. Finally, new treatment methods, including photothermal and magnetic hyperthermia, can also benefit from living cell-based delivery systems. The development of suitable biomaterials and biomedical engineering technology provides a favorable guarantee for the creation of new and effective cell-mediated drug delivery systems (Zhou et al., 2016; Sun, Liang, et al., 2017; Zhou et al., 2017). Herein, we describe the most common cellular delivery system, which is derived from various circulating cell types including RBCs, platelets, stem cells, and leukocyte. These systems have been processed to encapsulate different biologically active molecules, which can be used to carry drugs and particles into target diseases. These endogenous drug carriers offer a lot of advantages such as non-immunogenicity, biocompatibility, and natural targeting mechanisms. Therefore, they are made a suitable candidate for drug delivery. Furthermore, cell-based carriers have a longer life span in the circulation than synthetic carriers. They undergo apoptosis when they are aging or damaged, so they are completely biodegradable. Additionally, the cell carrier has a high loading capacity for cargo molecules due to their large volume.

Here, we focus on reporting the relevant information about different types of endogenous cells and examples of their use as drug carriers. In addition, three methods for loading drugs into cell carriers and the existing targeting strategies for various targets based on circulating cells are reviewed. In general, through the analysis of the data, the following conclusions can be drawn: endogenous cells are safe carriers that can release active therapeutic agents in the circulation. Therefore, they are made a suitable candidate for drug delivery. The various strategies for utilizing these endogenous cells discussed in this review reveal their effectiveness and further explore the numerous opportunities in this drug delivery field. The exploitation of cells as endogenous vectors is an interesting method for targeting many different disease states.

Although cell-mediated drug delivery systems have made great progress in improving diagnostic and therapeutic effects, there are still many challenges to the safety of clinical practice. First, the main challenge in the process of isolating cells *in vitro*, loading therapeutic agents, and reinjecting them back is to preserve the original properties of the cells. Second, drug loading efficiency may be lower due to the limited ability of immune surveillance and drug carriers to bind. A potential therapeutic agent that leaks from a carrier cell are another problem. Finally, because the mechanisms and development of many diseases are unclear, foreign cells with drug complexes may also exacerbate pathological conditions. As the most promising delivery system, carriers based on different endogenous cells still face some specific challenges, which may cause potential adverse effects. For RBCs, RBCs are an effective RES targeting system. This means that a large number of modifications are required to achieve effective delivery to other targets, which may lead to an inadvertent reduction of the biocompatibility of modified RBCs. Platelets are highly reactive and sensitive blood elements, which indicates that platelet-mediated delivery is related to the risk of affecting platelet function. Besides, for drugs that need to be loaded *in vitro*, the loading methods should be further studied to ensure that sufficient drugs are injected into the patient’s body in the platelets and are swallowed by macrophages. While for the stem cells, the challenge is still concerned with their genetic risk which may require further research. Compared with the other three carriers, the preparation and quality control of leukocyte are more difficult. Leukocyte-mediated delivery may risk overloading the RES and immune system, thereby hindering the host defenses. Furthermore, when too many carriers induce the activation of immune system components (such as phagocytes) and the release of pathological mediators including cytokines, ROS, etc., it may cause inflammation or aggravate inflammation.

Whatever these challenges, cell-mediated drug delivery has enormous potential to revolutionize current diagnostics and medical techniques. Advances in biomaterials may open up the new doors for DDS design. Combining living circulating cells and nanotechnology will become a new direction for controlling drug delivery while providing a powerful technology that can improve overall diagnosis and therapeutic outcomes. Thus, under a variety of pathophysiological conditions, studies of endogenous targeting are gaining momentum. Through the innovation of new technologies, the goal of improving and controlling drug delivery is achieved.
